# Impact of mutations in DNA methylation modification genes on genome-wide methylation landscapes and downstream gene activations in pan-cancer

**DOI:** 10.1186/s12920-020-0659-4

**Published:** 2020-02-24

**Authors:** Chai-Jin Lee, Hongryul Ahn, Dabin Jeong, Minwoo Pak, Ji Hwan Moon, Sun Kim

**Affiliations:** 1 Interdisciplinary Program in Bioinformatics, Seoul National University, Seoul, 08826 Korea; 2 Department of Computer Science and Engineering, Seoul National University, Seoul, 08826 Korea; 3 Bioinformatics Institute, Seoul National University, Seoul, 08826 Korea

**Keywords:** DNA methylation modifier, Sub-network clustering, DMR-DEG integration, Pan-cancer analysis, Genome-wide landscape

## Abstract

**Background:**

In cancer, mutations of DNA methylation modification genes have crucial roles for epigenetic modifications genome-wide, which lead to the activation or suppression of important genes including tumor suppressor genes. Mutations on the epigenetic modifiers could affect the enzyme activity, which would result in the difference in genome-wide methylation profiles and, activation of downstream genes. Therefore, we investigated the effect of mutations on DNA methylation modification genes such as DNMT1, DNMT3A, MBD1, MBD4, TET1, TET2 and TET3 through a pan-cancer analysis.

**Methods:**

First, we investigated the effect of mutations in DNA methylation modification genes on genome-wide methylation profiles. We collected 3,644 samples that have both of mRNA and methylation data from 12 major cancer types in The Cancer Genome Atlas (TCGA). The samples were divided into two groups according to the mutational signature. Differentially methylated regions (DMR) that overlapped with the promoter region were selected using minfi and differentially expressed genes (DEG) were identified using EBSeq. By integrating the DMR and DEG results, we constructed a comprehensive DNA methylome profiles on a pan-cancer scale. Second, we investigated the effect of DNA methylations in the promoter regions on downstream genes by comparing the two groups of samples in 11 cancer types. To investigate the effects of promoter methylation on downstream gene activations, we performed clustering analysis of DEGs. Among the DEGs, we selected highly correlated gene set that had differentially methylated promoter regions using graph based sub-network clustering methods.

**Results:**

We chose an up-regulated DEGs cluster where had hypomethylated promoter in acute myeloid leukemia (LAML) and another down-regulated DEGs cluster where had hypermethylated promoter in colon adenocarcinoma (COAD). To rule out effects of gene regulation by transcription factor (TF), if differentially expressed TFs bound to the promoter of DEGs, that DEGs did not included to the gene set that effected by DNA methylation modifiers. Consequently, we identified 54 hypomethylated promoter DMR up-regulated DEGs in LAML and 45 hypermethylated promoter DMR down-regulated DEGs in COAD.

**Conclusions:**

Our study on DNA methylation modification genes in mutated vs. non-mutated groups could provide useful insight into the epigenetic regulation of DEGs in cancer.

## Background

DNA mutation is one of the major causes of many diseases, thus understanding impact of mutations in genes is an important research problem. For example, mutations in oncogenes and tumor suppressor genes have been extensively studied over the years [1–3]. Some class of genes, e.g., epigenetic genes, have roles in cancer proliferation by modifying the epigenetic status of a cell, then the epigenetic status change affects gene expression regulation then cancer phenotype. Epigenetic genes are divided into three functional groups: epigenetic modulators, modifiers, and mediators [4]. Epigenetic modulators transmit signals to epigenetic regulators. Upon receiving such signal, epigenetic modifiers modify the epigenetic status of a genome. In response to the changes in the epigenome, epigenetic mediators then could change their biological roles. In addition, abnormal mutations in the epigenetic genes can adversely affect this epigenetic system, causing tumors.

Among epigenetic genes, DNA methylation related epigenetic modifiers, DNMT1, DNMT3A, MBD1, MBD4, TET1, TET2, and TET3, have been studied related to cancer [5–16]. DNMT3A mutation was found at a high rate of 22.1 percent of acute myeloid leukemia patients [17]. In our study, mutations in DNA methylation modifier genes were found in about 13 percent (1,474/11,315) of cancer patients from The Cancer Genome Atlas (TCGA) projects [18].

In general, mutations on a gene can affect the function of a gene, even loss or gain of a function. Many DNA methylation modification genes are enzymes. Thus, mutations on the epigenetic modifiers could affect the activity of epigenetic modifiers, which would result in the difference in genome-wide methylation profiles and in turn, activation of downstream genes. However, there is no systematic study on this important topic. In this paper, we investigated the effect of mutations on DNA methylation modification genes such as DNMT1, DNMT3A, MBD1, MBD4, TET1, TET2, and TET3 through a pan-cancer analysis. First, we investigated the effect of mutations in DNA methylation modification genes on genome-wide methylation profiles in 12 major cancer types in TCGA.

As a result, we found that genome-wide methylation landscapes were significantly different between two sample groups with mutations and without mutations in the DNA methylation modifier genes. Second, we investigated the effect of DNA methylations in the promoter regions on downstream genes in 12 cancer types. To investigate the effect of mutations on gene expression further, we chose an up-regulated gene cluster where differentially expressed genes (DEGs) were mostly hypomethylated promoter regions in acute myeloid leukemia and another down-regulated gene cluster where DEGs had mostly hypermethylated promoter regions in colon adenocarcinoma.

## Methods

### TCGA data of DNA methylome and transcriptome

To perform pan-cancer data analysis, we downloaded data for 12 major cancer types from TCGA: bladder cancer (BLCA), breast cancer (BRCA), colon adenocarcinoma (COAD), glioblastoma (GBM), head and neck squamous carcinoma (HNSC), kidney renal carcinoma (KIRC), acute myeloid leukemia (LAML), lung adenocarcinoma (LUAD), lung squamous carcinoma (LUSC), ovarian cancer (OV), rectal adenocarcinoma (READ) and uterine corpus endometrial carcinoma (UCEC). A total of 3,644 samples that had both methylome and transcriptome data were collected. Among 3,644 samples, 580 samples had at least one or more mutation in seven DNA methylation modifier genes, and 432 mutations except for synonymous mutation samples were finally identified. Thus samples were divided into two groups, one with mutations in DNA methylation modifiers (432 samples) and the other group (3,212 samples). Among 12 cancer types, OV type had no mutation sample. Thus, we analyzed 11 cancer types (Table 1).

**Table 1:** Number of samples per 12 major cancer type in TCGA

Cancer type	Total samples	Synonymous mutation samples	Non-synonymous mutation samples
TCGA-BRCA	784	14	47
TCGA-HNSC	521	19	41
TCGA-LUAD	455	14	66
TCGA-BLCA	408	24	70
TCGA-LUSC	369	16	55
TCGA-KIRC	319	3	17
TCGA-COAD	279	33	54
TCGA-UCEC	173	19	37
TCGA-LAML	170	0	33
TCGA-READ	93	3	8
TCGA-GBM	64	2	4
TCGA-OV	9	1	0
Total	3644	148	432

Each value represents the number of samples that have both methylome and transcriptome data and the number of samples that have mutations in seven DNA methylation modifier genes

### DEG analysis

The mRNA-seq data labeled by “illuminahiseq rnaseqv2 RSEM genes normalized” were downloaded from the firebrowse website (http://firebrowse.org/). A Bioconductor (version 3.8) EBSeq package [19] was used for the DEG analysis of RNA data. For each cancer type, we divided the samples into two groups into mutated versus non-mutated samples and performed DEG analysis. Number of DEGs was counted with false discovery rate (FDR) less than 0.05. Fold change values of gene expression level were used in the following clustering analysis.

### DMR analysis

The methylation data labeled by ”humanmethylation450 within bioassay data set function” were downloaded from the firebrowse website. For the methylation data analysis, the DMR was analyzed with a FDR of 0.05 using “bumperhunter” in the minfi package [20] of Bioconductor (version 3.8). For each cancer type, we divided the samples into two groups into mutated versus non-mutated samples as same as DEG analysis. The DMRs found were annotated using “matchgene” to select the genes with DMR in the promoter.

### Random sample test

Random sampling was performed to compare the seven DNA methylation modifier mutation samples of each cancer types. Random samples were selected with the same size as the seven DNA methylation modifiers mutation samples, and DEG and DMR analysis were performed 10,000 times using the selected and remaining samples.

### Log ratio of average methylation levels in promoter regions

To compare the methylation levels of each promoter region between the samples of which the seven DNA methylation modifier genes were mutated and the other samples, we firstly calculated the average of methylation levels of each promoter region for the samples with mutation and the other samples, respectively. After that, the log2 ratio of the averaged methylation levels was calculated and the equation is shown below:

$${LR}_{ij}={log}_{2}\frac{{Avg\_mut}_{ij}+pseudo}{{Avg\_non}_{ij}+pseudo}$$

where j indicates each probe, i is the index of cancer, *Avg*_*mut*_*ij*_ is the average of the methylation levels of probe j for the samples with mutation in cancer i, *Avg*_*non*_*ij*_ is the average of the methylation levels of probe j for the samples without mutation in cancer i and *LR*_*ij*_ is the log2 ratio of two average values of probe j in cancer i. Pseudo is the value of 0.001 we added to the averages to avoid the error caused by dividing by zero.

### Gene expression correlation analysis

For transcriptome data, correlation values between genes were calculated using Pearson’s correlation of “pearsonr” of scipy for each cancer type. The final correlation value between the final genes was calculated using the weight value of PPI score of STRING database. These correlation values are used the following clustering analysis.

### Graph-based clustering

We used igraph package [21] of R to detect multilevel community and perform sub-network clustering. For the graph-based clustering, we used the fold-change value of the gene and correlation values between genes. Before clustering, we discard genes with fold-change less than 0.2 and edge of correlation with less than 0.5. After clustering, we perform the GO enrichment test and one-sample t-test for each cluster.

### Network visualization with cytoscape

Visualization of the sub-network cluster is shown using Cytoscape (version 3.7.1).

### Promoter binding TF search by TRANSFAC

To search all TFs to bind the promoter sequence of DEG, we used TRANSFAC.

## Workflow

**Fig. 1:**
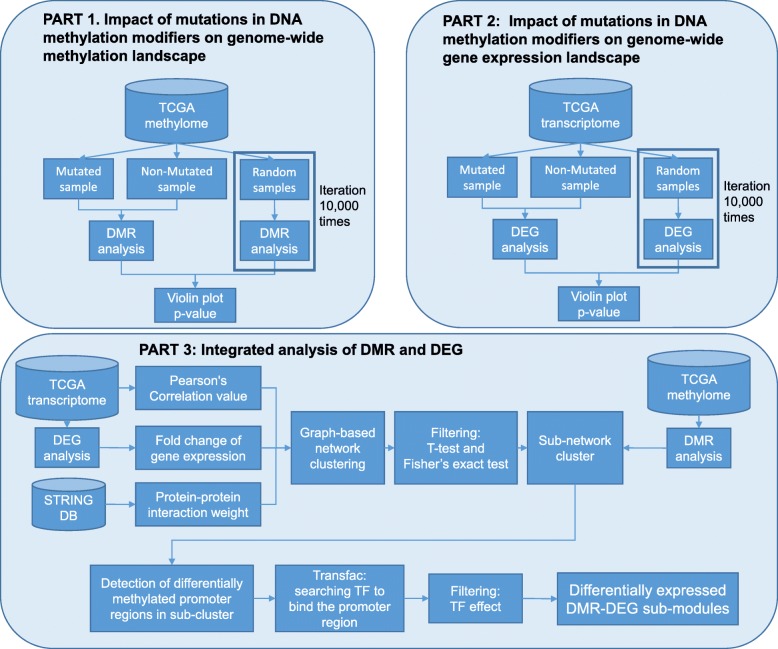
Workflow. See the “Workflow” section for more details

The analysis of the mutation data of seven DNA methylation modifiers on the pan-cancer scale was performed in three phases and the analysis workflow is shown in a schematic diagram (Fig. 1). In this section, the analysis process is briefly explained to help understand the analysis results. Detailed analysis methods are written in the “Methods” section.

### PART 1: impact of mutations in DNA methylation modifiers on genome-wide methylation landscape

First, we investigated the effect of mutations in DNA methylation modifiers on genome-wide methylation profiles.

#### 1-1. statistics on mutations in seven DNA methylation modifiers

Before investigating the genome-wide effects of seven DNA methylation modifiers, it was confirmed the distribution of 7 methylation modifier mutations in the mutation samples. Mutation frequencies in DNA methylation modifiers were collected for each cancer.

#### 1-2. genome-wide methylation landscapes

To investigate the genome-wide effects of seven DNA methylation modifiers, we analyzed the difference in DNA methylation profiles in pan-cancer. To compare the difference in methylation of samples that were divided into DNA methylation modifiers mutation, mutated and non-mutated samples (432 vs. 3,212 samples) in terms of log2 ratios (See “Methods” section for the detail).

#### 1-3. statistics of the number of differentially methylated regions (DMRs) between two groups

To confirm the effect of unbalanced samples and to evaluate whether these differences are significant or not, we analyzed them statistically. We compared the number of DMRs in samples with mutations in the DNA methylation modifier with the number of DMRs in randomly selected unbalanced samples. The analysis of DMR counts was performed with randomly sampled the same size as the number of mutation samples and repeated 10,000 times to calculate the *p*-value.

### PART 2: impact of mutations in DNA methylation modifiers on genome-wide gene expression landscape

Since DNA methylation can have significant effect on gene expression profiles, we compared gene expression profiles between the mutated and the non-mutated samples. In this part, we only compared gene expression profiles between two groups, without attempting to investigate the effect of DNA methylation on gene expression, which was reported in Part 3.

#### 2-1. statistics on gene expression profiles

DEG counts was collected from randomly chosen same size samples, repeating 10,000 times to calculate *p*-values.

#### 2-2. clustering analysis of transcriptome

To investigate biological functions of DEGs, we divided DEGs into smaller gene sets based on network based gene clustering analysis and then performed gene ontology (GO) term enrichment test on each set of DEGs to compare the difference in functions of genes between the mutated and non-mutated groups. Before performing sub-network clustering, correlation values between genes were calculated. Pearson’s correlation value was calculated for transcriptome data, and protein-protein interaction (PPI) score from STRING [22] database was multiplied by weight. Using the log2 fold-change value obtained from the DEG analysis, we removed genes that had opposite interaction or the small change amount. Thus, we selected a set of gene with over 0.15 of absolute value of log2 fold change of gene expression and over 0.5 positive correlated genes network. We performed graph-based sub-network clustering using iCluster (see “Methods” section) with fold change of gene expression using pre-processed gene-gene interaction score. To select meaningful clusters after clustering, we performed one sample t-test with gene expression levels and Fisher’s exact test using GO term enrichment test. Clusters with *p*-value under 10^−9^ was selected.

### PART 3: integrated analysis of DMR and DEG

Now, we tried to associate DEGs and DMRs between the two groups as below.

#### 3-1. integration of gene expression and methylation expression

To investigate the effect of DMRs on DEGs, we focused on methylation difference in the promoter regions. First, we selected gene clusters with significantly enriched DEGs and DMRs using a Fisher’s exact test for each of gene clusters. Then, gene sets were selected by considering negative correlation between promoter methylation and the corresponding gene expression.

#### 3-2. transcription factor (TF) binding site search with TRANSFAC

In addition to negative correlation between promoter methylation and the corresponding gene expression, we considered expression levels of TFs that could bind to the promoter regions. Thus, we searched for all TF binding sequences in the DEG promoter region using TRANSFAC [23].

#### 3-3. comparison without TF effect

Expression level of the TFs that had binding sites in the promoter regions was considered to remove cases where gene expression difference could result from TF expression difference. For example, if TF binding to the promoter of up-regulated DEG is not up-regulated, the up-regulated DEG can be determined by the effect of DMR regardless of the effect of TF. Thus, both up-regulated DEG with up-regulated TF and down-regulated DEG with down-regulated TF were removed.

## Results and discussions

### Part 1 - statistic analysis of mutation effect of seven DNA methylation modifier genes

To analyze the effects of seven DNA methylation modifier genes, we collected 3,644 TCGA methylome and transcriptome data. First, the number of mutation samples in DNA methylation modifier genes was found to be between 5% and 21% of the total sample for 11 major cancer types (Table 2). Excluding OV without mutation samples, 11 cancer types were analyzed.

**Table 2:** Summary of mutation status of seven DNA methylation modifier genes in each cancer

Cancer type	Total samples	Mutated samples	Non-mutated samples	Mutation sample ratio	Number of DMRs	Number of DEGs
TCGA-BRCA	784	47	737	6%	20,390	99
TCGA-HNSC	521	41	480	8%	21,442	57
TCGA-LUAD	455	66	389	15%	7,886	288
TCGA-BLCA	408	70	338	17%	12,711	379
TCGA-LUSC	369	55	314	15%	45,441	415
TCGA-KIRC	319	17	302	5%	23,544	127
TCGA-COAD	279	54	225	19%	26,491	535
TCGA-UCEC	173	37	136	21%	44,743	1,494
TCGA-LAML	170	33	137	19%	28,215	439
TCGA-READ	93	8	85	9%	58,058	205
TCGA-GBM	64	4	60	6%	77,753	215

Each value represents the number of samples that have both methylome and transcriptome data, the number of samples that have mutations in seven DNA methylation modifier genes, the number of samples that don’t have mutations, ratio of the mutation samples per non-mutation samples, the number of DMRs and the number of DEGs that were selected by 0.05 false discovery rate

**Fig. 2:**
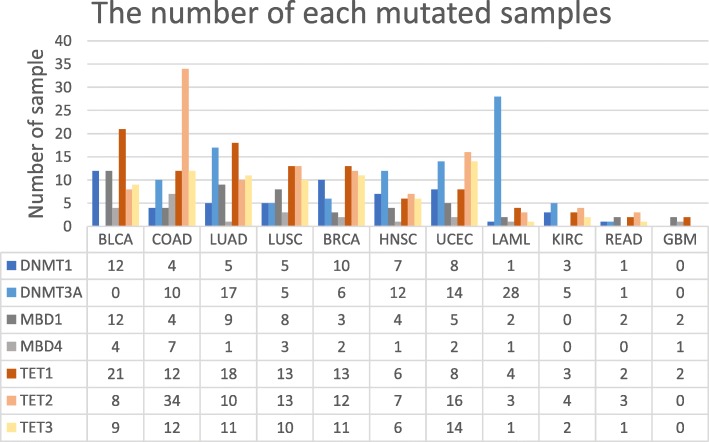
The number of samples that each of the seven DNA methylation modifier genes is mutated. A sample with mutations in multiple DNA methylation modification genes was counted redundantly as multiple genes. DNMT3A mutation is dominant in LAML samples. In COAD, mutations in TET1, TET2 and TET3 are dominant

The seven DNA methylation modifier genes that we studied were DNMT1, DNMT3A, MBD1, MBD4, TET1, TET2 and TET3. DNMT1 and DNMT3A function as DNA methyl-transfer and TET1, TET2 and TET3 have demethylation functions. Mutation statistics of the seven modifiers are summarized in (Fig. 2). Cancer types of BLCA, BRCA, COAD, LUAD, and LUSC were predominantly mutated in the TET genes that have demethylation functions. In the case of LAML, DNMT3A mutation samples were high, while remaining GBM, HNSC and KIRC, the ratio was similar. In the case of GBM, KIRC, and READ, the total mutation rate was less than 9%, and the number of mutations for each gene was 5 or less (Table 2). We should individually analyze to find a functional difference for each methylation modifier genes because the methylation modifier functions include methyl-transfer function and de-methylation that are opposite functions. However, since the number of samples is so small that it is very difficult to find a meaningful analysis result by each gene analysis, we first analyzed the global impact on the methylation dysfunction and then analyzed in depth. In addition, in the case of GBM and READ, the number of samples was eight or four, which makes it difficult to determine the representative characteristics of mutant cancers.

#### Effect of mutations in seven DNA methylation modifier genes on genome-wide methylation landscapes

**Fig. 3:**
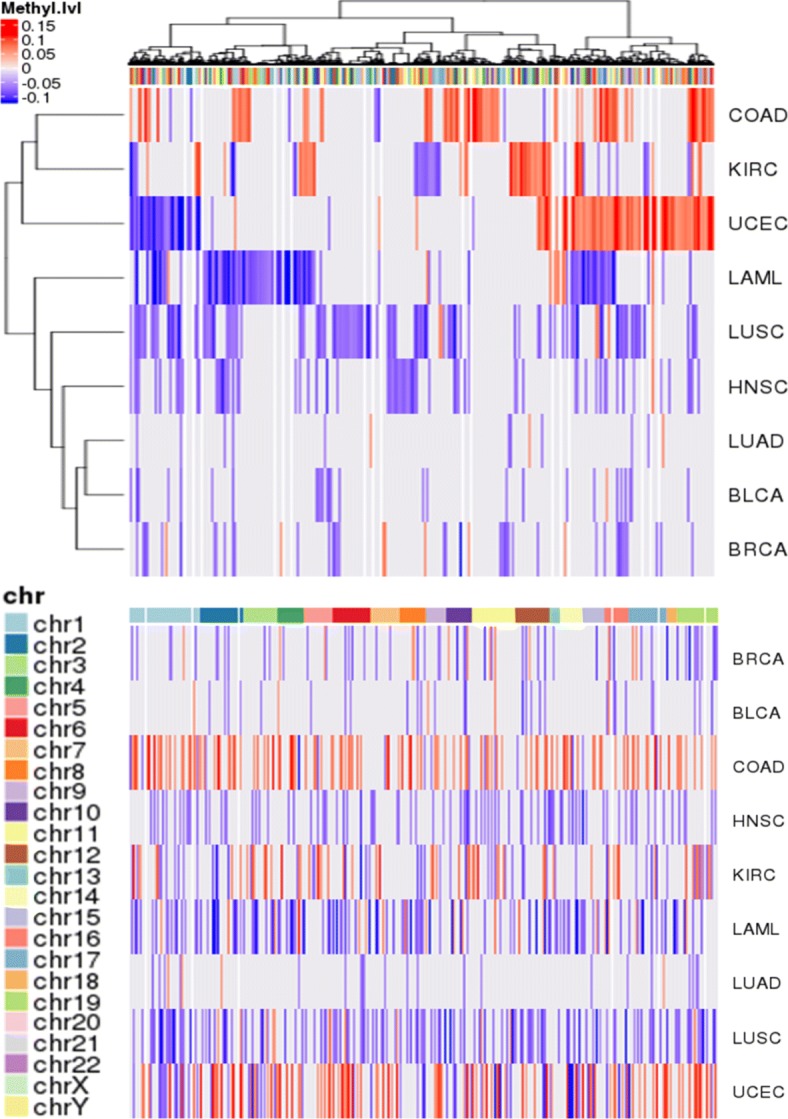
Genome-wide landscape of promoter methylation. Differential methylation level of gene promoter regions are profiled for 9 cancer types: bladder cancer (BLCA), breast cancer (BRCA), colon adenocarcinoma (COAD), head and neck squamous carcinoma (HNSC), kidney renal carcinoma (KIRC), acute myeloid leukemia (LAML), lung adenocarcinoma (LUAD), lung squamous carcinoma (LUSC), and uterine corpus endometrial carcinoma (UCEC). 9,580 genes showed hyper-methylation (red) or hypo-methylation (blue) in the promoter regions for at least one cancer type. In the lower panel, genes (i.e., column of the figure) are ordered according to the chromosomal position, and cancer types (i.e., row of the figures) are sorted by lexicographic order. In the upper panel, genes and cancer types are clustered in terms of methylation profile similarity

We compared genome-wide methylation landscapes between the mutated and the non-mutated groups. Since comparison of genome-wide methylation landscapes between the two groups was difficult to interpret, we compared promoter regions instead. Among the annotated 450,000 CpG sites, we selected the 140,040 sites as promoters when the sites are annotated as TSS200 or TS1500; TSS200 is the region that covers zero to 200 bases upstream of the transcription start site (TSS) and TSS1500 covers 200 to 1500 bases upstream of the TSS. For each of nine cancer types, methylation differences in 140,040 promoter regions of CpG sites were examined separately. We compared mutated and non-mutated samples of seven DNA methylation modifier genes, and the methylation values for each CpG site were expressed as log2 ratio values by comparing mean values. For the selected CpG sites, the average of DNA methylation of the mutation versus non-mutation samples was calculated as the log2 ratio and a heatmap was drawn by selecting 29,879 CpG sites with the log2 ratio value bigger than 1 or smaller than -1. Hypermethylated promoter is shown in red and hypomethylated promoter is shown in blue (Fig. 3). We measured the number of hyper-/hypo-methylated promoters in each cancer and estimated odd ratios and *p*-values of Fisher’s exact test. Each was calculated by applying different cutoff criteria for log2 fold changes of hyper-/hypo-methylated promoters (Table 3). In the heatmap results, COAD and UCEC have a large number of hypermethylated promoters, while LAML, LUSC, HNSC, BRCA, and BLCA have a large number of hypomethylated promoter regions. COAD showed the highest positive ratio and LAML had the most hypo-methylated promoter even when the cut-off criterion was raised. The heatmap results showed that there was a change in methylation due to the mutation of seven DNA methylation modifier genes, and detailed analysis was conducted to investigate the CpG site of promoter region with methylation changes in nine cancer types.

**Table 3:** Number of hyper-/hypo- methylated promoter in each cancer

Cancer	Hypermethylated promoter			Hypormethylated promoter			log2 odd ratio			*p*-value		
	0.05	0.075	0.1	0.05	0.075	0.1	0.05	0.075	0.1	0.05	0.075	0.1
COAD	2,923	921	246	348	41	3	3.0702	4.4895	6.3575	0	1.15E-201	1.E-67
UCEC	2,886	1,329	565	1,343	441	129	1.1036	1.5914	2.1308	1.20E-89	4.52E-88	5.E-62
KIRC	1,747	458	100	1,235	226	38	0.5003	1.0190	1.3959	2.52E-16	5.03E-18	1.E-07
LAML	407	73	15	3,274	1,285	446	-3.0079	-4.1377	-4.8940	0	5.86E-258	2.E-107
LUSC	297	27	1	3,882	886	94	-3.7082	-5.0362	-6.5545	0	2.33E-208	4.E-27
HNSC	63	2	0	2,500	284	15	-5.3104	-7.1497	-infinity	0	1.95E-80	6.E-05
BRCA	310	18	1	1,496	201	27	-2.2707	-3.4811	-4.7548	3.10E-159	8.28E-40	2.E-07
BLCA	87	3	0	1,031	60	3	-3.5668	-4.3219	-infinity	1.21E-187	8.09E-15	2.E-01
LUAD	183	8	2	521	17	0	-1.5094	-1.0874	+infinity	3.97E-36	0.107299	5.E-01

#### DMR analysis to investigate mutation effects of seven DNA methylation modifiers.

Mutated samples of seven DNA methylation genes were compared with non-mutated samples using bumperhunter of minfi package for DMR analysis. The significance of the number of DMRs potentially caused by the mutation of seven DNA methylation modifiers was compared with the number of DMRs in random samples. Random sampling DMR analysis was performed by repeatedly choosing samples of the same size for 10,000 times. *P*-value of the mutant sample was calculated from the distribution of DEG and DMR values obtained from 10,000 repeated tests. In the result of DMR test, 8 cancer types of 11, as BRCA, HNSC, LUAD, BLCA, LUSC, COAD, UCEC and LAML, showed significantly low *p*-value (Aditional file 1: Figure S2). The other cancer type, KIRC, READ and GBM, were not significant due to have few mutation samples (See Fig. 2). Overall, it seemed that mutations of seven DNA methylation modifier genes affected genome-wide promoter methylation differences.

### Part2 - genome-wide association analysis of mutation effect of seven DNA methylation modifier genes

#### Sub-network clustering result in pan-cancer scale

We performed graph-based clustering of DEGs. First, we used the network topology of STRING database and chose edges between two genes only when expression values of the two genes were highly correlated. Edges were weighted by the STRING database confidence scores. After that, the clustering was performed and the clusters were filtered using t-test.

**Fig. 4:**
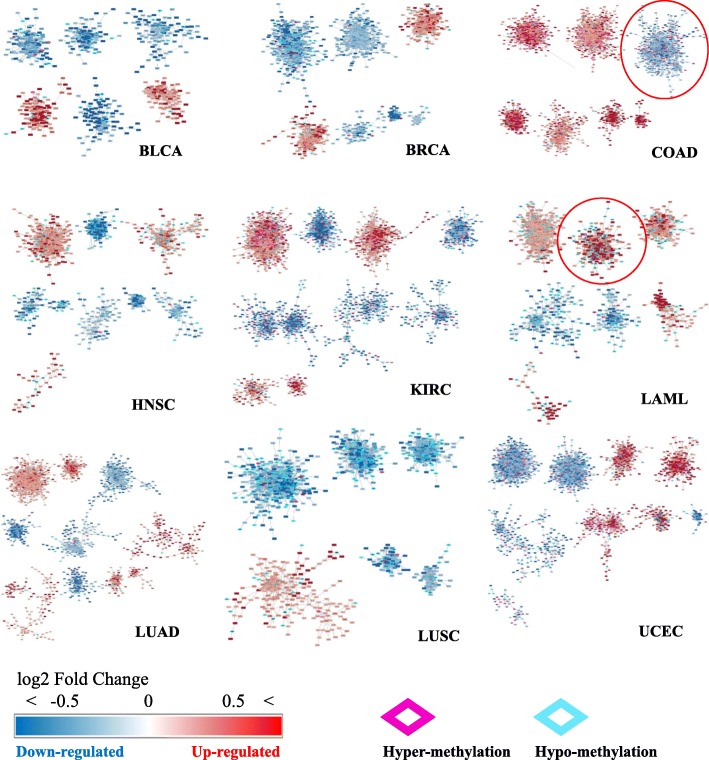
Graph-based clustering results. Up-regulated DEGs are colored in red, and down-regulated DEGs are colored in blue. The diamond borders of the genes are colored in pink or sky blue when the promoters of the genes are either hypermethylated or hypomethylated, respectively. The red circles indicate the selected clusters in LAML and COAD

The selected clusters were visualized using Cytoscape [24] (Fig. 4). Up-regulated DEG is displayed in a gradual red color and down-regulated DEG is displayed in a gradual blue color by the fold change value of gene expressions. Promoter DMR information was integrated into the DEG clusters and the case of DMR in the promoter of the up- and down-regulated DEG was marked in the cluster. DEGs with methylated promoter regions were colored in pink for hypermethylation and sky blue for hypomethylation.

#### Cluster selection for in-depth analysis

We performed Fisher’s exact test with the number of DMR-DEGs (differentially expressed gene with differentially methylated promoter region) in each cluster to select statistically significant clusters.

**Fig. 5:**
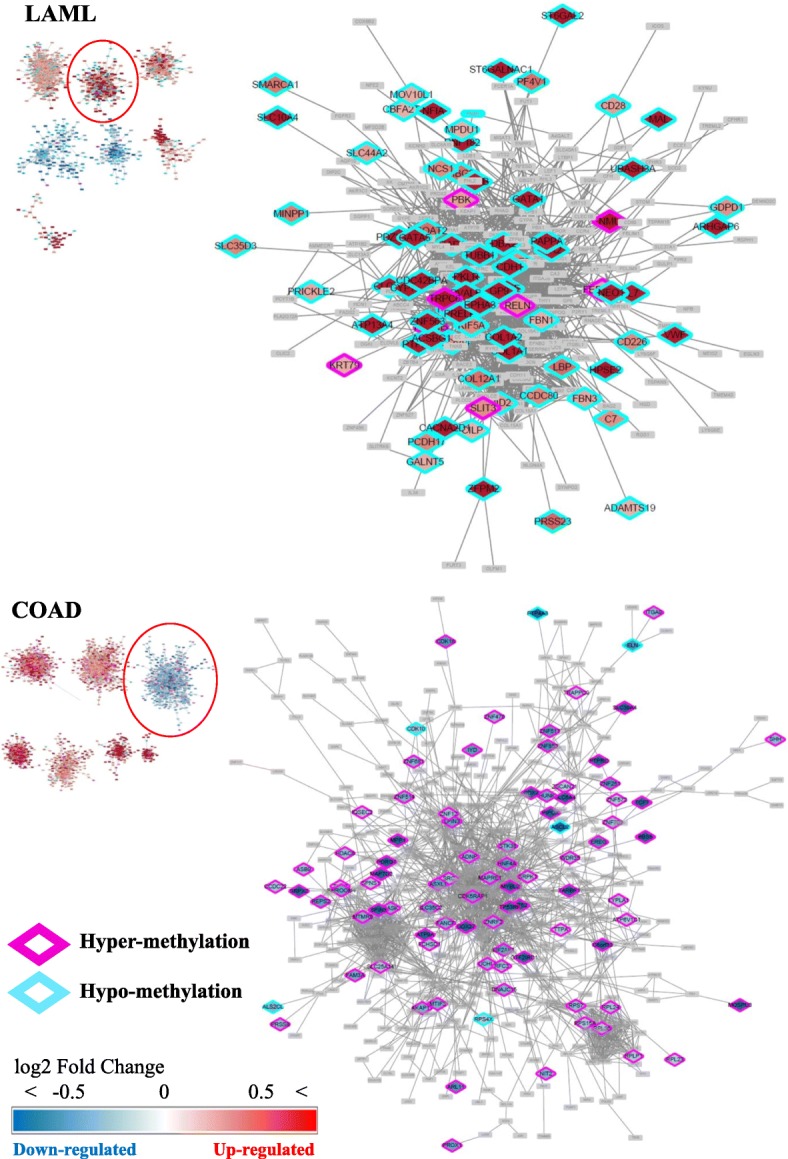
Selected sub-network clusters in LAML and COAD. Up-regulated genes were colored in red, and down-regulated genes were colored in blue according to the expression fold change level. DEGs without a differentially methylated promoter is shown in translucent gray. The borders of the genes are colored in pink or sky blue when the promoters of the genes are either hypermethylated or hypomethylated, respectively

A cluster in LAML was selected in which mutated samples of DNMT3A were abundant and DEGs were up-regulated. There were four clusters with up-regulated genes with hypo-methylated promoter, and one cluster containing genes with large log2 fold change of expression level was selected. In COAD clusters, TET1/2/3 genes were mutated with promoter hypermethylated, so we selected a cluster that contained the largest number of down-regulated DEGs. In the case of COAD, the most significant cluster with the highest number of DMR-DEG was selected. For the functional analysis of DEGs in the clusters, we selected a cluster of up-regulated DEGs in LAML and a cluster of down-regulated DEGs in COAD (Fig. 5).

#### TF selection related with DMR-DEGs

Among the genes in the clusters of COAD and LAML, we selected DEGs that the expression changes were not associated with TFs. To investigate TF-DNA-methylation interaction, we searched for all TF binding sties in the promoter regions using TRANSFAC [23] database. In COAD, there were 86 DMR-DEGs and we detected 170 TFs. In LAML, 75 DMR-DEGs were selected, and 179 TFs were detected by TRANSFAC using a promoter sequence of DEGs.

### Part 3 - DMR-DEGs in-depth analysis

#### Selection of cancers for in-depth analysis.

For the in-depth analysis to investigate the effect of mutations in DNA methylation modifiers, we first selected cancers based on the mutation profiles Fig. 2. In COAD, the number of the samples of which the demethylation-related genes, TET1, TET2 and TET3, were mutated was bigger than that of the samples with mutations in the methylation-related genes. On the contrary, in LAML, mutations in the methylation-related genes, e.g., DNMT3A, were dominant. We also looked genome-wide promoter methylation landscape to see relations between the mutations in the methylation-related genes and the methylation status of the promoters of the genes. As shown in Fig. 3, we were able to observe that there was a distinct signature of promoter hypermethylation in COAD (Fig. 5). On the contrary, in LAML, the promoters were hypomethylated rather than hypermethylated. GBM also showed the promoter hypomethylation but the number of samples with mutations was too small to analyze the effect of mutations (Fig. 2). Thus, we selected COAD and LAML for further analyses.

#### Selection of DMR-DEG possibly without TF-mediated regulation.

Before associating DMR-DEG, we excluded the DMR-DEGs that the expression changes were possibly affected by TFs. Among selected TFs that had binding sites in the promoter regions (see cluster selection in PART 2), if expression levels of TFs were different significantly between the mutated and non-mutated sample groups, TF expression difference could affect expression levels of downstream genes, thus we remove genes whose promoter regions had binding sites of such TFs. We set 0.2 and -0.2 as cutoff values for *log*_2_ fold change to determine if a gene or a TF is up-regulated or down-regulated. When a gene is up-regulated and a TF targeting the gene is up-regulated, the DEG was removed. Likewise, when a gene is down-regulated and a TF targeting the gene is also down-regulated, the DEG was removed. Finally, 54 DMR-DEGs in LAML and 45 DMR-DEGs in COAD were selected and studied for functional effects (Table 4).

**Table 4:** List of 54 DMR-DEGs in LAML and 45 DMR-DEGs in COAD

Selected cluster	DMR-DEGs
Cluster of LAML	ACSBG1, ARHGAP6, ATP13A4, C7, CACNA2D1, CBFA2T3, CCDC80, CD226, CD28, CILP, COL12A1, COL1A1, COL1A2, DBX2, EPHA3, FBN1, FBN3, FERMT2, FN3K, GATA1, GATA5, GDPD1, GFI1B, GP9, HBG2, HPSE2, IL7, KIF5A, LBP, LRRC8E, MAL, MBOAT2, MINPP1, NCS1, NEO1, NID2, NLGN1, NMU, PAPPA, PF4V1, PKLR, PRELP, PRICKLE2, PTPRR, RNF182, SLC35D3, SLC44A2, SLIT3, ST6GAL2, ST6GALNAC1, TUBB1, VWF, ZFPM2, ZNF563
Cluster of COAD	ADNP, AKAP11, ARL11, ASB9, ASXL1, ATP9A, BBS5, C8orf33, CASK, CDK18, DNAJC15, FCHSD1, GTF2IRD1, HDAC8, HUNK, IFT52, IQSEC2, IYD, LYPLA1, MOSPD3, MPP1, MRPS33, MTIF3, NIT2, PIPOX, PRSS8, REPS2, RPL24, RPLP1, RPS7, SHH, SLC25A14, SPIN3, SPNS1, TARBP1, TP53RK, TTPA, UCHL3, WDR35, ZNF12, ZNF251, ZNF514, ZNF517, ZNRF2, ZXDB

#### up-regulated DEGs related with hypo-DMR in LAML

54 up-regulated DEGs with hypomethylated promoters were selected in LAML. To investigate the biological function of these genes, we searched the literature to find relevance of these genes to LAML. For 54 DEGs in LAML, we searched with the terms “methylation” or “acute myeloid leukemia”. CACNA2D1, CBFA2T3, CD226, EPHA3, GATA1, GFI1B, IL7, NMU, PTPRR, SLIT3 and ST6GAL2 genes are related with disorder of methylation in LAML. CACNA2D1 (Voltage-dependent calcium channel subunit alpha-2/delta-1) encodes a member of the alpha-2/delta subunit family, a protein in the voltage-dependent calcium channel complex. CACNA2D1 has DMR in oxytocin signaling pathway in LAML [25].

CBFA2T3 is known to operate via a fusion gene mechanism with INADL and TM2D1 in AML [26].

CD226 (Cluster of Differentiation 226, DNAM-1 (DNAX Accessory Molecule-1)) is a 65 kDa glycoprotein expressed on the surface of natural killer cells, platelets, monocytes and a subset of T cells. TIGIT binding with CD226 is up-regulated on CD8(+) T cells in LAML [27]. EPHA3 (ephrin type-A receptor 3) has been implicated in mediating developmental events, particularly in the nervous system. Receptors in the EPH subfamily typically have a single kinase domain and an extracellular region containing a Cys-rich domain and 2 fibronectin type III repeats. EphA3 was methylated in leukemia patients [28]. GATA1 (GATA-binding factor 1) regulates the expression of an ensemble of genes that mediate the development of red blood cells and platelets. Its critical roles in red blood cell formation include promoting the maturation of precursor cells. GATA-1 binds to the PU.1 gene and inhibits expression in LAML [29]. IL7 (Interleukin 7) stimulates proliferation of all cells in the lymphoid lineage (B cells, T cells and NK cells). IL-7 has abnormal methylation in peripheral blood of LAML patients [30]. GFI1B (Growth factor independent 1b, Zinc finger protein Gfi-1b) are highly expressed in LAML [31].

NMU induced specifically acute promyelocytic leukemia in Sprague-Dawley rats [32]. PTPRR has been recently identified as a fusion partner of the ETV6 gene in AML patients bearing an inv(12)(p13q13) and leads to GM-CFS-independent STAT3 activation [33].

SLIT3 (Slit homolog 3 protein) is a ligand-receptor SLIT-ROBO family. Low expression of SLIT and high expression of ROBO1 and ROBO2 suggests their participation in LAML pathogenesis [34].

ST6GAL2 was detected with unique DMR gene for AML subtype [35].

SLC44A2 is related with LAML. SLC44A2 (Choline transporter-like protein 2) is located in a pathway controlling DNA damage and repair, and affects the survival in LAML [36].

In GO-term enrichment test with “blood coagulation”, “cell adhesion”, “platelet activation”, “extracellular matrix organization”, “cellular response to transforming growth factor beta stimulus”, “response to stimulus”, “collagen fibril organization”, “multicellular organismal process”, “response to endogenous stimulus”, “skin morphogenesis” and “cell activation” (Table 5).

**Table 5:** Enriched GO terms of 54 DMR-DEGs in LAML

GO-term ID	term description	gene count	FDR
GO:0007596	blood coagulation	9 of 288	0.0002
GO:0007155	cell adhesion	13 of 843	0.0002
GO:0030168	platelet activation	6 of 120	0.00039
GO:0030198	extracellular matrix organization	8 of 296	0.00045
GO:0071560	cellular response to transforming growth factor beta stimulus	5 of 140	0.0075
GO:0050896	response to stimulus	35 of 7824	0.0269
GO:0030199	collagen fibril organization	3 of 39	0.0269
GO:0032501	multicellular organismal process	31 of 6507	0.0272
GO:0009719	response to endogenous stimulus	12 of 1353	0.0298
GO:0043589	skin morphogenesis	2 of 9	0.0416
GO:0001775	cell activation	10 of 1024	0.0446

#### Down-regulated DEGs related with hyper-DMR in COAD

45 down-regulated DEGs with hypermethylated promoters were selected in COAD. To investigate the biological function of these genes, we searched the literature to find relevance of these genes to COAD. For 45 DEGs selected in the cluster of COAD, we searched the literature with the terms “methylation” or “Colon adenocarcinoma”. HDAC8, HUNK, PRSS8, RPS7 and UCHL3 genes are related with disorder of methylation in COAD.

HDAC8, one of the histone deacetylase (HDAC) family of transcriptional co-repressors, has emerged as important regulators of colon cell maturation and transformation [37]. Abnormal changes in DNA methylation level of HUNK were found in tumor tissues of patients [38]. PRSS8 acts as a tumor suppressor by inhibiting Sphk1/S1P/Stat3/Akt signaling pathway [39].

RPS7 (40S ribosomal protein S7) is a component of the 40S subunit. In eukaryotes, ribosomes, the organelles that catalyze protein synthesis, consist of a small 40S subunit and a large 60S subunit. Aberrant promoter hypermethylation of RPS7 inhibits colorectal cancer growth [40].

UCHL3, a member of the ubiquitin C-terminal hydrolase family, has a similar activity to UCHL1 and is ubiquitously expressed in various tissues. Methylation of the UCHL3 promoter CpG island was completely unmethylated in the colorectal cancer [41].

ADNP, ASB9 and NIT2 genes are related with COAD. ADNP is a repressor of WNT signaling in colon cancer [42]. Low ASB9 expression have higher malignant potential, such as cell invasiveness and liver metastasis resulting in a poor prognosis for human colorectal cancer [43].

NIT2 (Nitrilase Family Member 2) has a omega-amidase activity to remove potentially toxic intermediates by converting alpha-ketoglutaramate and alpha-ketosuccinamate to biologically useful alpha-ketoglutarate and oxaloacetate. Downregulation of NIT2 inhibits COAD cell proliferation and induces cell cycle arrest [44].

SHH and WDR35 genes are related with abnormal methylation in cancer. The increased and constitutive SHH expression is implicated in gastric carcinogenesis, and that promoter methylation may be an important regulatory mechanism of SHH expression [45]. WDR35 has functions in cell signaling and apoptosis. The methylation levels of WDR35 was consistent with an inverse relationship with the mRNA expression levels in a large number of ALL cells [46].

In GO-term enrichment test with “Biological Process” category, the 45 genes in COAD were found to be related with “cytoplasmic translation”, “ peptide biosynthetic process”, “SRP-dependent cotranslational protein targeting to membrane”, “cotranslational protein targeting to membrane”, “protein targeting to ER”, “translation”, “viral gene expression”, “nuclear-transcribed mRNA catabolic process, nonsense-mediated decay” and “viral transcription” (Table 6).

**Table 6:** Enriched GO terms of 45 DMR-DEGs in COAD

GO-term ID	term description	gene count	FDR
GO:0002181	cytoplasmic translation	3 of 55	0.0002
GO:0043043	peptide biosynthetic process	4 of 175	0.0006
GO:0006614	SRP-dependent cotranslational protein targeting to membrane	3 of 90	0.001
GO:0006613	cotranslational protein targeting to membrane	3 of 94	0.0012
GO:0045047	protein targeting to ER	3 of 98	0.0013
GO:0006412	translation	4 of 233	0.0018
GO:0019080	viral gene expression	3 of 111	0.0019
GO:0000184	nuclear-transcribed mRNA catabolic process, nonsense-mediated decay	3 of 113	0.002
GO:0019083	viral transcription	3 of 114	0.0021

## Conclusions

### High-dimensional feature space data analysis using sub-network clustering

Determining DEGs affected by methylation changes is a research problem of dealing with high dimensional feature spaces that need s to combine gene expression levels and methylation expression levels. Our approach to dealing with this challenging problem was to use a network based approach.

Clustering genes by combining protein-protein interaction scores and gene correlation values were effective in identifying DEGs clusters that could be affected by DNA methylation modifiers. In addition, we considered TF-DNA interference by DNA methylations in the promoter regions to focus more on the effect of mutations in DNA methylation modifiers only. Many of the genes that were identified by our approach have been shown to be related to cancer development in the literature regarding the effects of methylation. Some genes that were determined in this study are also likely to be related to cancer expression by methylation, which could be good testable hypotheses for additional biological experiments.

### Biological meaning and functions of the identified DMR-DEGs

Recently, the effects of epigenetic changes in phenotypic changes including disease developments have been investigated extensively. However, the effects of mutations in epigenetic modifiers have not been well studied so far. To investigate how epigenetic changes are acquired, it is very important to investigate biological mechanisms that could cause epigenetic changes. In this context, we investigated the effects of mutations in DNA methylation modifier genes on the transcriptomic profiles in samples of mutated vs. non-mutated DNA methylation modifiers in the pan-cancer scale. We identified 54 DEGs affected by seven DNA methylation gene mutations in LAML patient samples and 61 DEGs in COAD patients. Gene expression levels of these genes increased (DEGs of LAML) or decreased (DEGs of COAD) without potential effects of TFs that could bind to the promoter regions of the genes. In other words, differences in methylation status in the promoter regions of the genes could be the main reason why these genes were expressed differentially. 28 of 33 mutant samples in LAML had mutations in DNMT3A, and 34 of the 54 samples in COAD had mutations in TET2. Mutations in DNMT3A could result in hypomethylation in the promoter regions due to abnormal methyl transfer, resulting in increased gene expression. Mutations in TET2 could result in hypermethylation in the promoter regions of genes due to abnormalities in demethylation function. In the clusters of LAML, 10 of 54 DMR-DEGs, that has highest number of DMR, were known to be associated with LAML in the literature. 7 of the 10 genes were associated with abnormal methylation with LAML in the literature. In case of COAD, 8 of 45 DMR-DEGs were associated with COAD and 4 genes among the 8 genes were associated with abnormal methylation in COAD in the literature. In this study, we reported that these genes are likely to be related to the development of cancer due to changes in DNA methylation. However, functional impact and biological interpretation of our findings are yet to be confirmed although we provided GO term enrichment analysis and related papers in the literature. As we have more samples available, our approach could contribute to elucidating testable hypotheses on the roles of mutations in DNA methylation modifiers.

## Supplementary information

**Additional file 1** Figure S1 Case ratio of 7 DNA methylation modifiers mutations in each TCGA 33 projects. Figure S2 Violin plot result of DMR analysis.

## Abbreviations

DEG: Differentially expressed gene
DMR: Differentially methylated region
DNA: Deoxyribonucleic acid
GO: Gene ontology
RNA-seq: Whole transcriptome sequencing
RNA: Ribonucleic acid
STRING: Search tool for the retrieval of interacting genes/proteins
TCGA: The cancer genome atlas
TF: Transcription factor
