# Supplementary material for: Impact of mutations in DNA methylation modification genes on genome-wide methylation landscapes and downstream gene activations in pan-cancer
Source: BMC Med Genomics. 2020 Feb 24;13(Suppl 3):27. doi: 10.1186/s12920-020-0659-4 (PMC7038532; doi:10.1186/s12920-020-0659-4)
Supplement: Supplementary file 1 — Additional file 1 Figure S1 Case ratio of 7 DNA methylation modifiers mutations in each TCGA 33 projects. Figure S2 Violin plot result of DMR analysis. [file 12920_2020_659_MOESM1_ESM.pptx]

## Slide 1
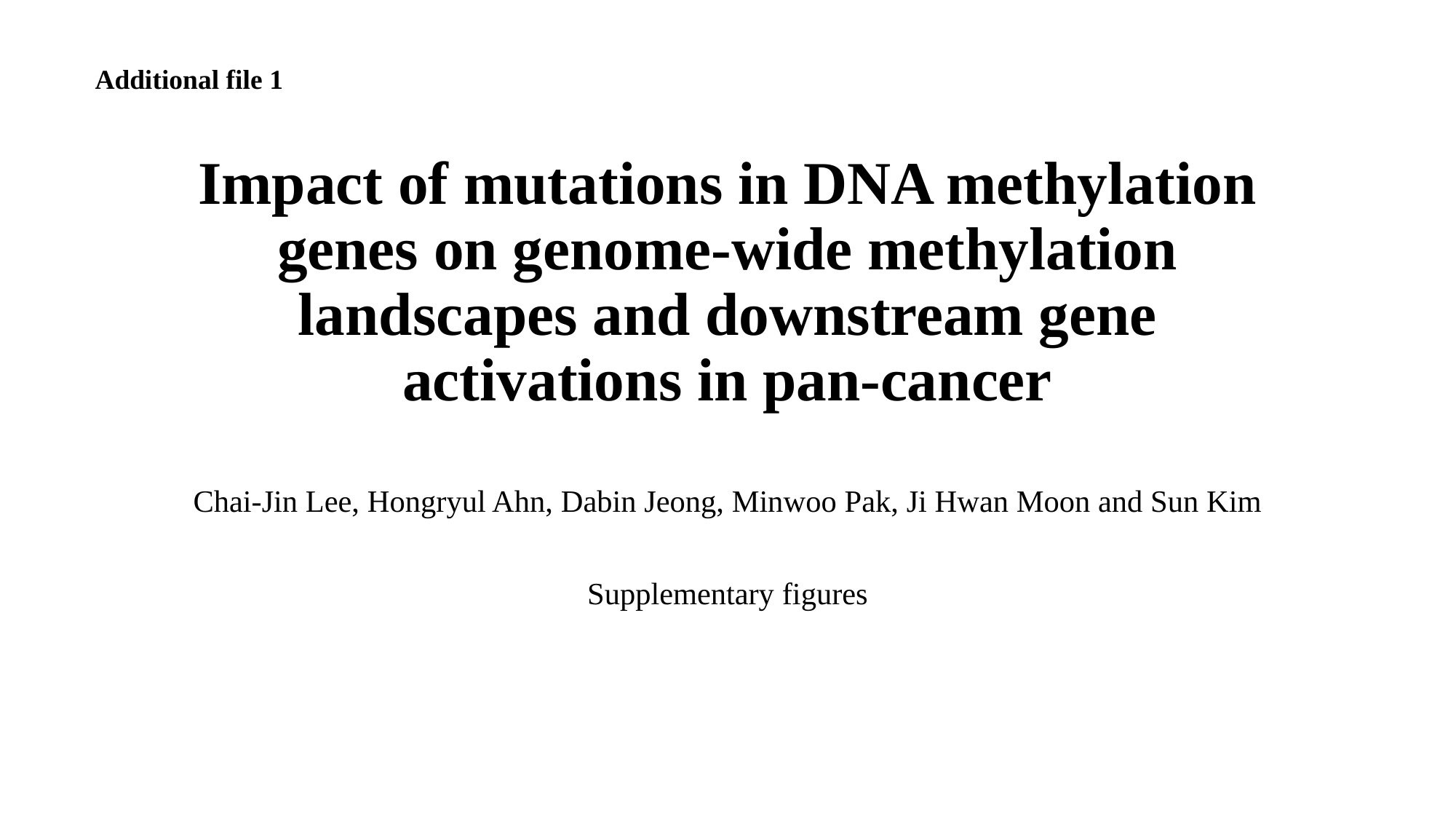

Additional file 1
# Impact of mutations in DNA methylation genes on genome-wide methylation landscapes and downstream gene activations in pan-cancer
Chai-Jin Lee, Hongryul Ahn, Dabin Jeong, Minwoo Pak, Ji Hwan Moon and Sun Kim
Supplementary figures

## Slide 2
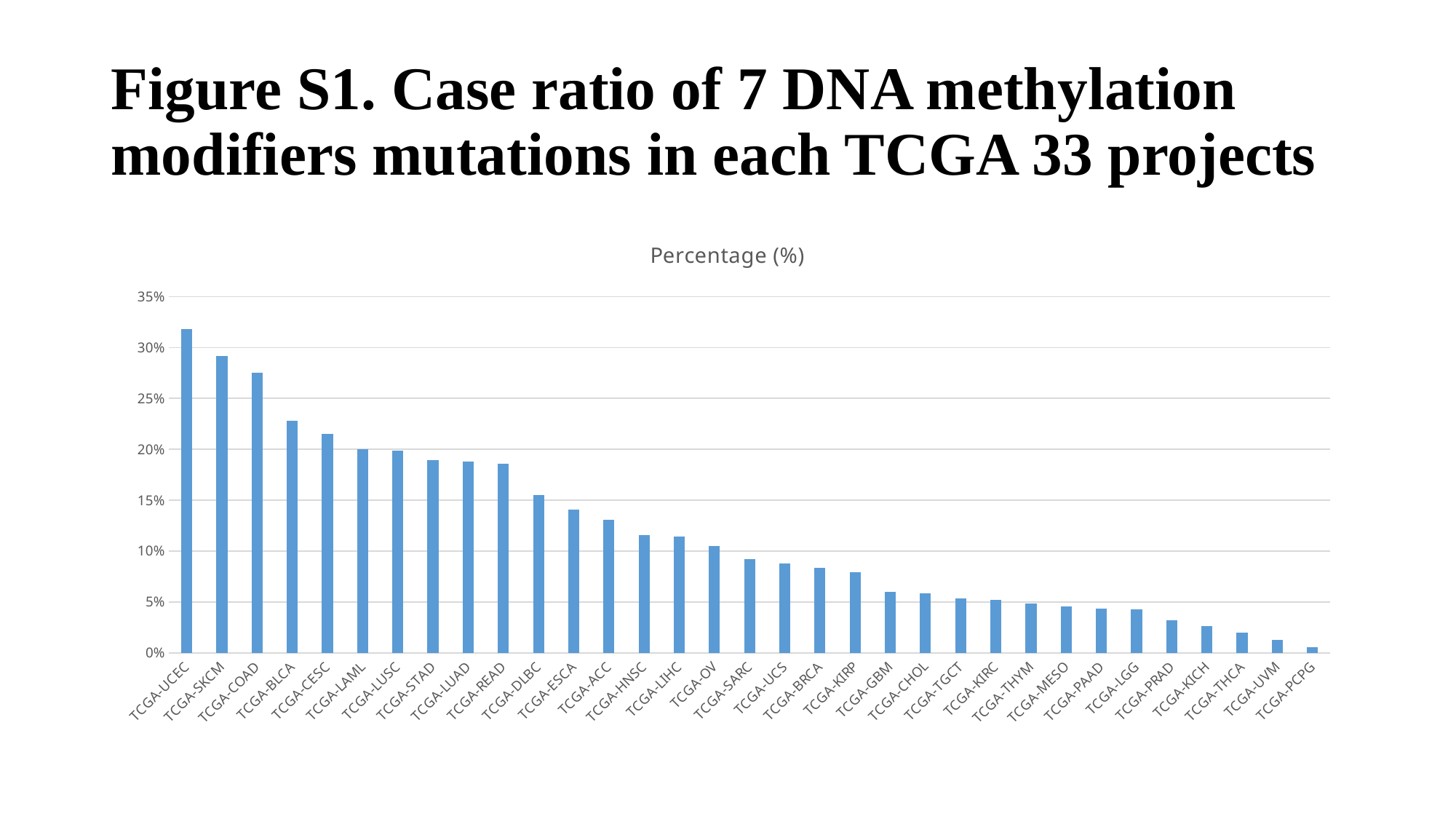

# Figure S1. Case ratio of 7 DNA methylation modifiers mutations in each TCGA 33 projects
### Chart: Percentage (%)
| Category | percentage |
|---|---|
| TCGA-UCEC | 0.31785714285714284 |
| TCGA-SKCM | 0.29148936170212764 |
| TCGA-COAD | 0.2754880694143167 |
| TCGA-BLCA | 0.22815533980582525 |
| TCGA-CESC | 0.21498371335504887 |
| TCGA-LAML | 0.2 |
| TCGA-LUSC | 0.1984126984126984 |
| TCGA-STAD | 0.18961625282167044 |
| TCGA-LUAD | 0.18803418803418803 |
| TCGA-READ | 0.18604651162790697 |
| TCGA-DLBC | 0.15517241379310345 |
| TCGA-ESCA | 0.14054054054054055 |
| TCGA-ACC | 0.13043478260869565 |
| TCGA-HNSC | 0.11553030303030302 |
| TCGA-LIHC | 0.11405835543766578 |
| TCGA-OV | 0.10526315789473684 |
| TCGA-SARC | 0.09195402298850575 |
| TCGA-UCS | 0.08771929824561403 |
| TCGA-BRCA | 0.08378870673952642 |
| TCGA-KIRP | 0.07903780068728522 |
| TCGA-GBM | 0.059967585089141004 |
| TCGA-CHOL | 0.058823529411764705 |
| TCGA-TGCT | 0.05333333333333334 |
| TCGA-KIRC | 0.0521415270018622 |
| TCGA-THYM | 0.04838709677419355 |
| TCGA-MESO | 0.04597701149425287 |
| TCGA-PAAD | 0.043243243243243246 |
| TCGA-LGG | 0.04263565891472868 |
| TCGA-PRAD | 0.032 |
| TCGA-KICH | 0.02654867256637168 |
| TCGA-THCA | 0.01972386587771203 |
| TCGA-UVM | 0.0125 |
| TCGA-PCPG | 0.00558659217877095 |

## Slide 3
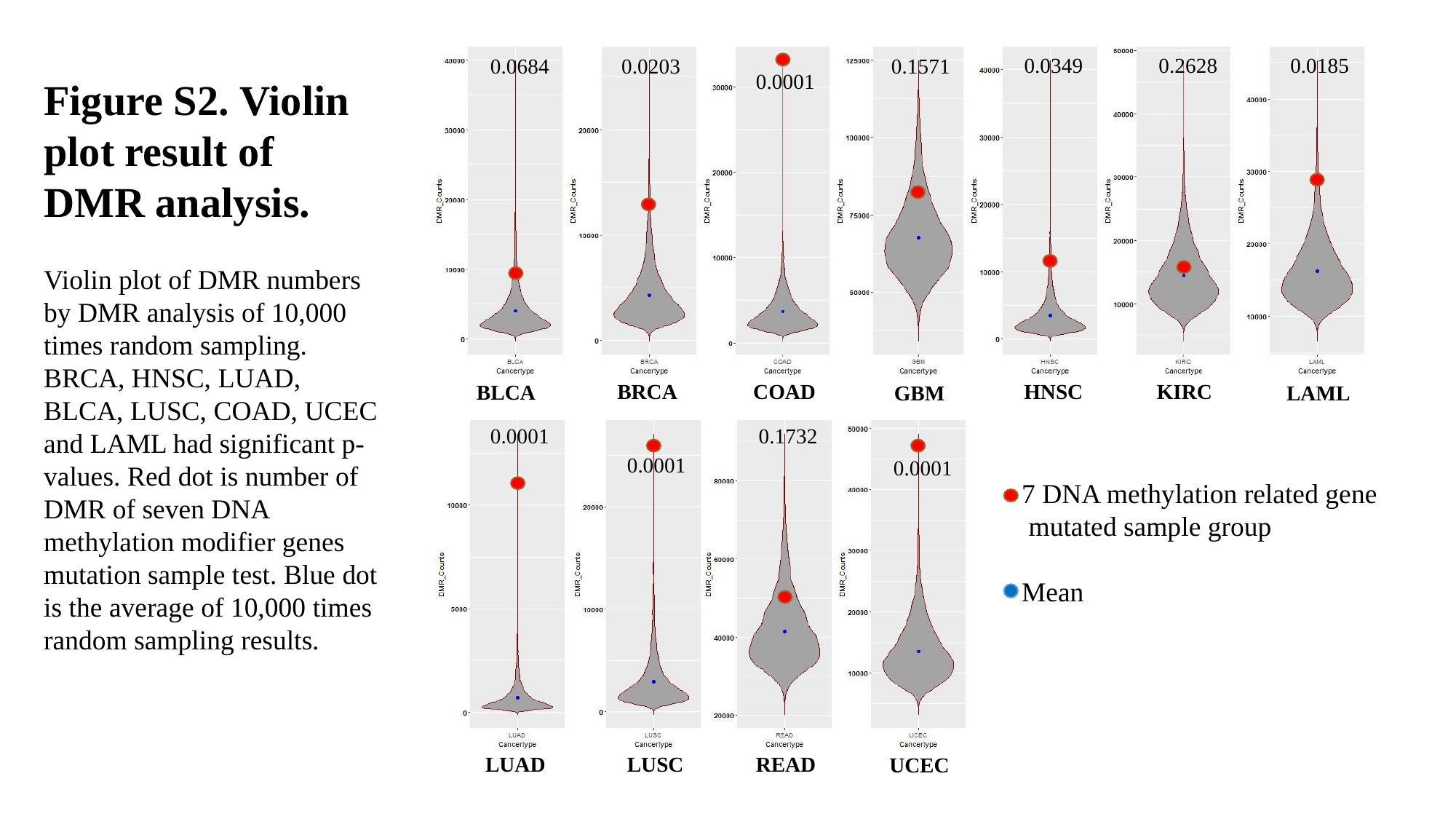

0.2628
0.0185
0.0349
0.0684
0.0203
0.1571
0.0001
COAD
HNSC
KIRC
BRCA
BLCA
LAML
GBM
0.0001
0.1732
0.0001
0.0001
7 DNA methylation related gene
 mutated sample group
Mean
READ
LUSC
LUAD
UCEC
Figure S2. Violin plot result of DMR analysis.
Violin plot of DMR numbers by DMR analysis of 10,000 times random sampling. BRCA, HNSC, LUAD, BLCA, LUSC, COAD, UCEC and LAML had significant p-values. Red dot is number of DMR of seven DNA methylation modifier genes mutation sample test. Blue dot is the average of 10,000 times random sampling results.
